# Patterns in the skin microbiota differ in children and teenagers between rural and urban environments

**DOI:** 10.1038/srep45651

**Published:** 2017-03-31

**Authors:** Jenni Lehtimäki, Antti Karkman, Tiina Laatikainen, Laura Paalanen, Leena von Hertzen, Tari Haahtela, Ilkka Hanski, Lasse Ruokolainen

**Affiliations:** 1 Department of Biosciences, University of Helsinki, FI-00014 Helsinki, Finland; 2 National Institute for Health and Welfare, FI-00271 Helsinki, Finland; 3 Skin and Allergy Hospital, Helsinki University Central Hospital, FI-00029 Helsinki, Finland

## Abstract

The composition of human microbiota is affected by a multitude of factors. Understanding the dynamics of our microbial communities is important for promoting human health because microbiota has a crucial role in the development of inflammatory diseases, such as allergies. We have studied the skin microbiota of both arms in 275 Finnish children of few months old to teenagers living in contrasting environments. We show that while age is a major factor affecting skin microbial composition, the living environment also discriminates the skin microbiota of rural and urban children. The effect of environment is age-specific; it is most prominent in toddlers but weaker for newborns and non-existent for teenagers. Within-individual variation is also related to age and environment. Surprisingly, variation between arms is smaller in rural subjects in all age groups, except in teenagers. We also collected serum samples from children for characterization of allergic sensitization and found a weak, but significant association between allergic sensitization and microbial composition. We suggest that physiological and behavioral changes, related to age and the amount of contact with environmental microbiota, jointly influence the dynamics of the skin microbiota, and explain why the association between the living environment skin microbiota is lost in teenager.

Skin, the largest organ of the human body, mediates information from the outer world and provides the first line of defense against pathogens, toxins and hostile environmental conditions. This barrier-function-role is both physical and immunological and it is supported by microbial communities inhabiting the skin^1^. The skin microbiota contribute to the barrier function by competing with pathogens and, importantly, communicating with immune cells in the skin^2^, to modulate the local^3^ and systemic^4^ immune responses. The skin microbiota and immune mediators, such as the complement system^5^, have a two-way interaction, indicating that commensal microbes should be considered as an essential part of healthy skin. Furthermore, accumulating evidence suggests that the composition of microbiota, especially in the gut but also on the skin, can have major influence on individual health^6^,^7^. For example, the skin microbiota is altered in certain dermatological conditions, such as in psoriasis^8^ and in atopic dermatitis^9^, which further suggests the importance of skin microbial communities for human health.

As the microbial communities in our bodies are constantly open for colonization from the environment, it is important to understand how different features in human life contribute to the development and current state of human microbiota. The facts that the skin microbial communities resemble those in soils^10^, and that microbes present in air differ between land-use types^11^, suggest that the surrounding living environment could affect the composition of skin microbiota^12^. This is supported by observations that the local environment can shape gut microbial communities in people sharing similar lifestyle and diet^13^. The skin microbiota is likely to be more affected by the surrounding environment than that in the gut, due to a more immediate contact with the environment. While a recent study showed that the habitat has a strong influence on the skin microbiota in bats^14^, the relationship between the living environment and the composition of skin (and gut) microbiota is still not well understood. Moreover, the development of microbiota may differ in dissimilar microbial environments. This is potentially important as the early cross-talk between the developing immune system and microbiota can strongly affect the immune function in later life^15^.

In this study, we compared the skin microbiota of human subjects living in rural and urban environments, to see whether the microbial composition is affected by the living environment, and whether the development of microbiota is systematically different in rural and urban environments. We specifically focused on children (a less studied age group), as the microbial dynamics are especially interesting during the period of immune system development in early life. In addition, we assessed the similarity of microbial composition between symmetrical body sites, as this question has got little attention before. Moreover, we measured allergic sensitization of children as we also wanted to test the biodiversity hypothesis^16^,^17^, which states that the green living environment can support immune function through colonizing beneficial environmental microbiota. Our results suggest that the skin microbiota differs between rural and urban children, and that the size of this effect varies with age, eventually disappearing in teenagers. However, we found no clear association between the living environment, skin microbiota, and allergic sensitization. This is likely due to a discrepancy between the fact that the foundation of the immune tolerance is laid in early life^15^, and our results showing a strong age and environmental dependence off the skin microbial composition.

## Results

### The skin microbiota depends on age and living environment

**Figure 1:**
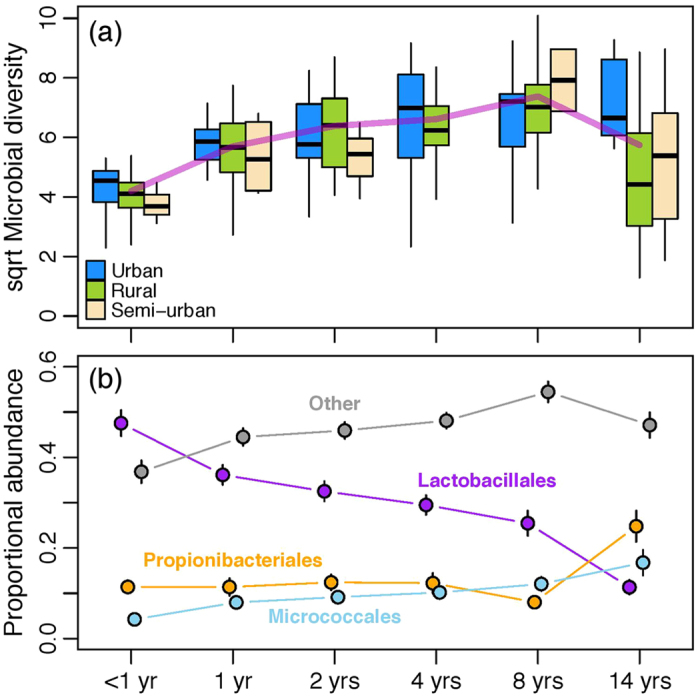
The diversity of skin microbiota in different age groups. (**a**) Diversity in skin microbiota tends to increases with age. Diversity was calculated with *q* = 1, which corresponds to Shannon’s index. Box colour indicates the living-environment and the purple line indicates the mean diversity for each age group. In the oldest age group, children living in rural area (*n* = 26) have significantly lower diversity than those living in Helsinki (Urban, *n* = 8): *P* = 0.018. (**b**) The increase in diversity during the first eight years of life is associated with a reduced dominance of the Order Lactobacillales (namely *Streptococcus*) and a relatively even increase in other taxa, whereas the reduction in diversity in puberty is due to Actinobacteria (such as *Propionibacterium acnes*) becoming dominant. Means (±SE) are given for each age group.

**Figure 2:**
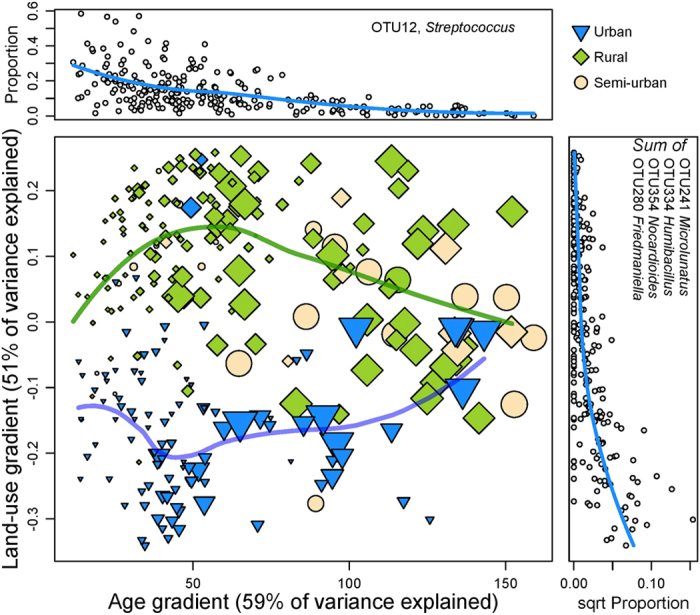
The composition of skin microbiota differs between urban and rural children. (Main panel) Predictions from random forest (RF) analyses plotted as an ordination. The x-axis shows the predicted age and the y-axis shows the predicted land-use around the current home. The variances explained indicate how well skin microbiota independently predicts either age or land-use. Symbol size indicates true age in months, symbol shape indicates the living environment of the current home (Urban, Rural, or Semi-urban), and the lines give spline fits to the respective groups. In the case a child had moved from the home at birth, the color of the symbol indicates the environment of the subject at birth. RF analyses were based on sqrt-transformed, CSS-normalised counts. RF analysis gives the OTUs which are the most important discriminators between subjects: The upper panel shows the relative proportion of OTU 12 (*Streptococcus*) across predicted age. The right panel shows the sum of proportions of several OTUs across the predicted land-use gradient. The segregation between living environments is not due to different variances along the land-use gradient for children of different age (Levene’s test: *F* = 0.45, *P* = 0.81), nor differences in sample size (X^2^ = 3.39, *P* = 0.64).

Age is a major determinant of skin microbial composition. The diversity of microbiota increases at least during the first eight years of life (Fig. 1a), which is associated with a reduced dominance of *Lactobacillales* (especially genus *Streptococcus*) on the skin (Fig. 1b, and the upper panel figure in Fig. 2). In 14-year old children there is greater inter-individual variation in diversity than in the younger age groups (Fig. 1a). At this age the relative abundance of *Lactobacillales* decreases and is replaced by other groups (namely *Actinobacteria* and *Proteobacteria*) (Fig. 1b) and the relatively large variation in diversity is mostly explained by the varying abundance of *Actinobacteria* such as *Propionibacterium* and *Corynebacterium* (Fig. S11). Also, the diversity is on average lower, as compared with eight-years-olds (*P* = 0.0019), which is due to children from more rural surroundings having lower skin microbial diversity (Fig. 1b). At the age of 14, the diversity in skin microbiota resembles closely that observed for adults (unpublished data). Moreover, the integrity of the skin microbial composition tends to be greatest in the youngest and the oldest age groups, while the rest are difficult to differentiate from one another (Fig. S8).

Next we asked whether the composition of the skin microbiota reflects subject age and living environment. Independent random forest analyses indicate that 59% of inter-individual variation in age, and 51% in land-use around the current home, can be explained by variation in skin microbiota (Fig. 2). In other words, the composition of the living environment and age of children clearly influence the composition of skin microbiota. As noted above, the age gradient is most clearly associated with a reduction in the abundance of *Streptococcus* (Fig. 2, upper panel), while the difference between rural and urban environments comes from increasing abundance of summed *Microlunatus, Humibacillus, Nocardioides* and *Friedmaniella* (Fig. 2, right panel, more information provided in Fig. S9).

The effect of the living environment is not the same across ages. Differences in land-use are best reflected in the microbiota of young children between one to four years of age (Fig. 2). In contrast, there is a slightly weaker signal of the living environment in newborns and in eight-year-old children while no signature of the living environment is seen in teenagers (Fig. 2). To quantify the unique and shared contributions of microbiota in predicting subject age and environmental land-use, we calculated a partitioning of prediction variance using partial least squares regression (PLS, Table S2). A total of 88% of variation in age plus land-use could be explained by variation in microbiota. The unique contribution to age was 26% and that to land-use was 0.5%, while the shared component was as high as 57%. As suggested by the RF analysis (Fig. 2), age is quite predictable (PLS: 83% of variation explained when considered alone), given the microbial composition of individuals. While land-use is also fairly well predicted from the microbiota (PLS: 62% of variation explained when considered alone), the predictability depends on age (as can be seen in Fig. 2), such that the prediction of land-use overlaps almost entirely with that of age.

**Figure 3:**
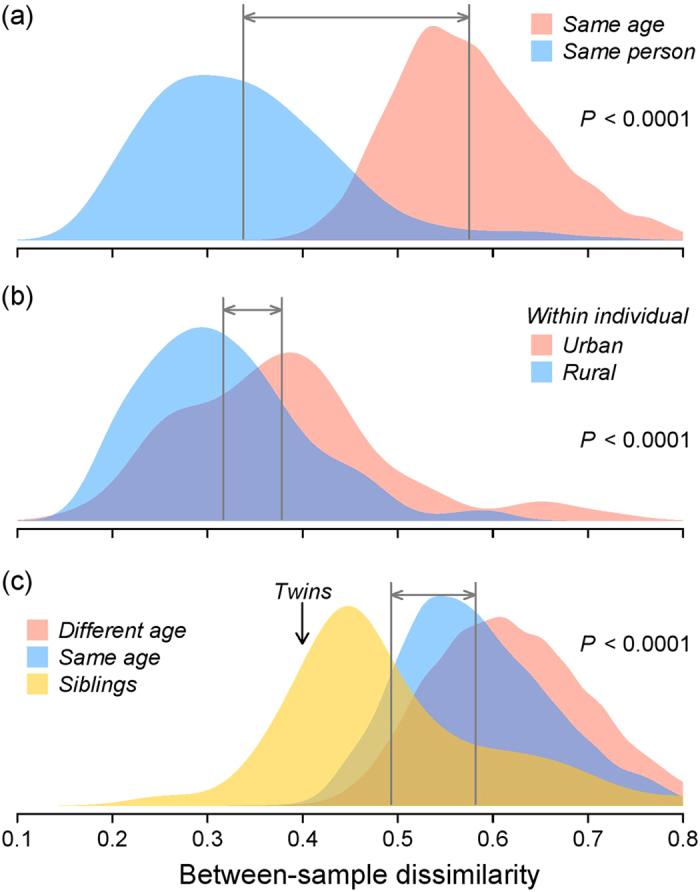
The between- and within-individual variation in the skin microbiota. The x-axis in figures shows the Bray-Curtis dissimilarity between compared samples, and grey vertical lines are the average values of given groups. In figures (**a**) and (**b**) samples from the dominant and non-dominant arms have been compared, while in figure (**c**) the dominant arms of different individuals have been compared. Figure (**a**) shows that the intra-individual dissimilarity between arms is significantly smaller than inter-individual dissimilarity. Figure (**b**) shows that the intra-individual dissimilarity between arms is significantly smaller in rural than in urban children. Figure (**c**) shows that the inter-individual dissimilarities are significantly smaller between sibling-pairs than in other pairs meaning that children share more of their microbiota with their children than with other children. Our data included one dizygotic twin-pair (teenagers of different sex), who shared more of their microbiota than sibling pairs on average, providing interesting example about the effect of shared microbiota.

We also considered within-individual variation in different ages and environments by comparing samples from symmetrical body sites (the dominant and the non-dominant arms). Not surprisingly, the samples from different arms in the same individual were significantly more similar than samples from different arms between individuals within the same age group (Fig. 3a). Moreover, the within-individual difference between arms increased with increasing age (Fig. S10). Interestingly, the skin microbiota of symmetrical sites was more similar in rural children than in urban children (Figs 3b and S10), except for 14 year-olds for which the intra-individual variation was higher in rural subjects (Fig. S10).

### Other factors contributing to variation in skin microbiota

We were able to trace many factors contributing to the composition of the skin microbiota. Our data had 86 children with at least one sibling enrolled to the study. Children living in the same household share more of their skin microbiota, which can be seen in higher between-sibling community similarity as compared with either pairs of children with same age or any pair of children (MRM, *P* = 0.001, Fig. 3c). Age and the composition of the living environment remain significant predictors of differences in microbial composition, even after controlling for potential confounding factors (Table 1). Also, the original library size of the sample, pet ownership, total number of siblings, gender and usage of antibiotics during the past year contribute to the total variation, but their influence is relatively small (total effect size, *R*^2^ = 0.055; Table 1).

We bring original library size to the analysis because sample size differs a lot in skin microbiota samples. The effect of original library size poses a potential problem (however, samples were normalized through all analyses). We do not know whether the variation in original library size is a technical artefact or reflects biologically relevant differences between subjects (for example the prevalence of Propionibacterium correlates with library size). Allergic sensitization to food allergens do not influence the variation in skin microbiota composition, but sensitization to inhalant allergens was weakly but significantly associated with variation in microbial composition (Table 1). We also tested the effect of diagnosed atopic dermatitis on skin microbiota with random forest analysis, but the classification of age- and sex-matched children to atopic dermatitis and healthy groups was not possible.

## Discussion

Our results indicate that the influence of age and the living environment on the composition of skin microbiota can be interdependent. The signal of environmental land-use around homes in skin microbiota is most prominent in toddlers (1–4 years of age), while its effect is less pronounced in babies (less than a year of age) and older children already in school (8 years of age). Finally, in adolescents (14 years of age), the imprint of the living environment in the skin microbiota is no longer detectable. Several features in child development, such as changes in skin physiology (further discussed later), can explain the observed interaction between environment and age. After birth, gut microbiota closely resembles that of the mother within the first year of life^18^,^19^. This indicates that newborns are mainly exposed to environmental microbiota indirectly, filtered by their parents. As children grow, they start to actively explore their surrounding environment. Growing-up is also related to the expansion the spatial scale of environmental exposure, and an increase in social contacts. These changes lead to a diminishing correspondence between the environment just around the home and the total exposure to environmental microbes. Teenagers, however, assumingly spend more of their time indoors than younger children as outdoor activities are a central part of the day in Finnish daycares and primary schools, but later time spent in these activities decreases. Increased indoor time can partly explain why the effect of surrounding environment disappears in teenagers, because indoor microbiota does not generally reflect that of outside^20^ (however, opposite was discovered in ref. 21), but rather composes from human-skin derived microbes^20^,^22^.

Skin microbial composition in different environments has been considered in only a few earlier studies. Ruokolainen *et al*.^23^ showed that the proportional abundance of *Proteobacteria* increases on the skin of healthy children from urban to green environment, which was not found in this study (*R*^2^ = 0.002, *P* = 0.54). Recently, it was reported that the skin microbiota differs between rural and urban Chinese, ranging between 12–60 years of age^12^. This study also reported a significant interaction between gender, age and residence (rural vs. urban) in affecting skin (Vf) microbial composition (using the same distance measure as in ref. 12, we did not get a significant interaction; *P* = 0.2). In contrast with our study, the disparity between urban and rural microbiotas remains in adolescent, and in adults. One potential explanation for this inconsistency is cultural differences; the urban and rural populations in China tend to have contrastingly dissimilar lifestyles^12^, whereas differences in lifestyle across rural-urban gradients are likely less dramatic in Finland. For example, our study does not include children from farms, but in study by Ying *et al*.^12^ most rural subjects had a traditional farmer lifestyle. Similar cultural differences, which should be accounted in microbiome research, are likely to arise elsewhere as well. In the US, children spend most of their time indoors irrespective of age^24^, whereas in Finland this tends to change across ages. Thus, the living environment is unlikely to affect the skin microbiota, if direct or indirect (e.g., via a dog: ref. 25) contact with that environment is lacking. These examples indicate that the interdependent effect of age and environment found here can be a product of cultural features rather than universal in all children. More work is clearly needed to better understand the patterns of microbial colonization in different cultural and environmental circumstances.

The comparisons between symmetrical skin sites are currently rare. Previous studies have shown that the within-individual dissimilarity of symmetric skin sites is smaller than between-individual variation^26^,^27^. While our results corroborate these earlier findings, our data also suggests that the amount of within-individual variability between arms can depend on the living environment; urban children tended to have more dissimilar arms than rural children (Figs 3 and S10). We were expecting to see more within- and between-individual variation in rural subjects as the environmental microbiota samples tend to be more heterogeneous in rural than in urban environments^28^. While the reason for this opposite finding in our study is unclear, a few plausible explanations can be given. For example, urban children are likely to be more frequently engaged in social contacts than rural children, diversifying skin microbiota overall. Also, the urban environment in Helsinki, having just 620 000 inhabitants, may differ from those studied in US (in ref. 28), and can provide more heterogeneous environment than rural environments elsewhere in Finland. Thus, yet again, cultural and national differences can partly explain the patterns discovered in this study. It is also possible that rural environment or lifestyle somehow promotes higher similarity between arms, so that the effect of dominance of arms becomes less clear. The other pattern discovered in comparison between arms was that dissimilarity tends to increase with age, which likely reflects the increasingly differing use of the dominant and non-dominant hands associated with child growth.

The skin microbiota is developing rapidly during the first year of life^29^, but little is known about later development. Although this is not a longitudinal study, our results also provide interesting insight into the succession of skin microbiota from newborn to adult-like. The diversity of the skin microbiota rises steadily until the age of eight years, most clearly during first two years, which indicates some differences in the timespan of maturation of gut^19^,^30^ and skin microbiota. While the increasing diversity is associated with decreasing dominance of *Lactobacillales* it is not possible to tell whether this results from changes in habitat quality or overall increase in microbial abundances with age. In teenagers, the diversity drops when compared to eight years old children supporting a previous study^31^. The characteristics of the skin habitat change considerably with age—thickness, oiliness, hair cover, etc.^32^—which naturally affects the habitat favorability to different bacterial taxa. In teenagers, puberty, and associated changes in the skin physiology, are likely important reasons for changes in the microbial community composition and diversity. Moreover, the use of skin care products, such as deodorants^33^, may increase in teenagers, which can shape the skin microbiota, but unlikely so on the skin region (Vf) studied here which is not that exposed to varied products. Puberty might cause a strong enough selection pressure to mask any influence of the living environment in teenagers (skin poses strong selection on environmental microbes in amphibians^34^). In addition to well-understood changes in the physio-chemical properties of the skin with age^32^—having a somewhat deterministic influence on the development of microbiota—microbial composition is likely to be affected by an individual’s ability to sample environmental microbiota as described earlier. For example, the relatively rapid convergence of the gut community, in comparison to that of the skin, could be understood via the prominent role of diet in shaping the gut microbial composition^35^. A child’s diet starts to resemble that of adults fairly quickly, usually just a few years after birth, whereas in case of the skin the microbial input likely continues to change also after school age due to several lifestyle-related factors.

In addition to the microbial samples, we also defined allergic sensitization by measuring the blood serum immunoglobulin E level and the incidence of allergic symptoms. The prevalence of sensitization to inhalant allergens was higher in rural than in urban younger children, but the difference was only marginally significant (1–4 years old, *P* = 0.053). In 8–14 years old children sensitization is more common in urban environments, even though this difference is not significant (*P* = 0.56). Moreover, in both young and old children, the prevalence of rhinitis symptoms is higher in urban environments (Table 2). While we were able to find both differing skin microbiota and prevalence of allergic diseases across areas, the link between skin microbiota and sensitization was weak (Table 1). We suggest that cross-sectional snapshot data is not sufficient for uncovering such association. This mismatch can be understood as follows. Many studies have indicated that the critical period for the cross-talk between the microbiota and the immune system is rather short in early childhood^15^,^36^. However, our results suggest that the skin microbiota reflects the current exposure milieu of an individual (Fig. 2), which means that the present microbiota can be completely different from that during the active development of the immune system. On the other hand, allergic sensitization, especially to inhalant allergens, is usually manifested after six years of age, when the levels of IgE have stabilized^23^. Thus, a connection between the composition of microbiota and realized health cannot be established from cross-sectional data, as either the health information is not meaningful (in young children), or, when sensitization can be reliably judged, the sampled microbial composition does not any longer reflect that at the time of critical exposure. Moreover, it is currently unclear whether the skin microbiota of any area of the body has a potential to have a systemic effect on human health^3^ even though its importance in local immunity has strong evidence^5^,^37^, and that mouse model results suggest that systemic effects are possible^4^.

There are several potential confounding factors that could have affected our results. For example, the results regarding the prevalence of allergic sensitization and symptoms across areas (Table 2) are somewhat conflicting when compared to earlier work, reporting increased prevalence of sensitization from rural to urban environments (for example, in Finland^23^,^38^). This indicates that our dataset is not fully representing the population, probably due to rather low participation rate (17%) and differences in willingness of guardians of healthy and symptomatic children to participate in the study. Also, even though the difference between Finnish urban and rural populations is likely to be smaller than between those populations in China, the background of the children differs considerably (Table 3). Thus, part of the difference between rural and urban populations can be explained by lifestyle rather than land-use. Additionally, the selected body site can influence the power different factors have in shaping the community^39^. For example, it has been suggested that environmental factors have a greater role in shaping microbial communities at sebaceous sites than at dry sites, such as the volar forearm studied here, because moist sites tend to better promote bacterial growth^40^. Finally, research methods are currently developing in microbiome research. Chosen methods can have affected our results, as suggested in Table 1, showing that also original library size of the samples can affect the composition of microbiota; whether this is artificial or biological is unclear.

We show that the composition of microbial communities depends on individual age and their living environment. The effect of living environment is strongest in young children. This is important given that the first years of life are most critical for immune system development in cross-talk with microbiota. Age-related skin physiology and lifestyle also contribute to microbial composition. We suggest that children growing-up in contrasting environments are exposed to dissimilar microbial environments potentially influencing their future health through the altered development of microbiota. Our findings suggest that future studies focusing on determinants of human microbiota, and the interaction between health and microbiota should put emphasis on the following. First, while experimental work is important for proof of principle, additional longitudinal studies on human subjects are needed for establishing causality. Second, even though living environment seems to be an important determinant of microbiota, more information is needed about the effect of different cultural habits and lifestyle related factors on environmental exposure. Third, the current way of describing microbial communities is overly simplistic. Strain-level differences are very individual-specific^41^,^42^, and thus it is interesting whether certain signature strains across geographical areas and land-use gradients exist. Lastly, the potential of natural environments as a source of beneficial microbes should be further addressed^43^. We conclude, that in studies focusing on human microbiota, a wide approach should be implemented, meaning that human microbiota is likely a gradient composing on several interacting but separate habitats, and these all are nested under the environment humans are living in^44^.

## Materials and Methods

### Data collection

Three study regions from Finland were selected for the study: *the city*-*center of Helsinki* (Urban), which is urbanized capital next to the Baltic Sea, *the city*-*center of Joensuu* (Semi-urban), which is a less urbanized city in eastern Finland with easy access to surrounding natural areas, and *rural areas around North*-*Karelia* (Rural) in eastern Finland characterized by a high coverage of agricultural land, forests, and lakes. Children were recruited either with invitation letters sent to randomly selected guardians (altogether 1530 letter sent as low participation rate was expected), or through a random sample of schools and child health clinics (around 150 children invited). In final data, we have 275 children (participation rate 17%) from dissimilar residential environments in Finland. Children enrolled were from 2 months to 14 years of age belonging to six different age groups (Table 4). Most children who were included in the study, had lived their whole life in the same apartment, or moved inside areas, which share similar environmental features. However, one child had born in rural area and then moved to urban environment while nine children had done the opposite. These children were not removed from analysis, so that effect of current and birth environment could be compared (Fig. 2). No further exclusion or inclusion criteria was included as the main focus on the study was to explore the differences in the skin microbiota between living environments, and we wanted to ensure sufficient number of subjects. In the final dataset, the gender distribution is even as well as the size of different age groups (Table 4).

Data collection was done by study nurses during winter season (November 2014-February 2015), who sampled enrolled children in sampling happenings organized in cities and towns around research area. Families were not instructed to diverge from their normal daily (such as washing) routines prior to examination. Nurses collected skin swab samples from the skin of both arms for analysis of skin microbiota. Sterile swabs (Floqswabs, Copan flock technologies) were moistened with a sterile solution of 0.15 M NaCl and 0.1% Tween 20. Samples were taken from 5 cm times 5 cm areas, or from slightly smaller areas in case of younger children, from volar forearm (Vf). Sampling area on the skin was swabbed several times from right to left, and from up to down with constantly rotating the swab. We chose to sample arms, as we expect them to be regularly in contact with the surrounding environments, and thus have the potential to discriminate rural and urban subjects. Sera were collected for analysis of allergic sensitization (specific immunoglobulin E analysis). Five millimeters venous blood from antecubital fossa was collected from children unless they were less than 12 months of age. In total, serum samples were obtained from 226 children.

Parents or guardians of the participating children answered to a questionnaire on internet (response rate 97%). We collected information about children’s allergic symptoms, parent’s symptoms, lifestyle, and living environment. Questions about allergic symptoms were based on those developed for the international study of asthma and allergies in childhood (ISAAC)^45^. Other questions included well-known allergy-related factors such as the length of breastfeeding, interaction with animals and number of siblings. Moreover, nature connection was searched with a large set of questions as the effect of living environment is the central focus in this study. Translated questionnaires can be found from the supplementary forms (Supplementary forms S1–S3).

### Laboratory analysis

#### Skin microbiota

After collection, the skin microbiota swab samples were immediately placed on dry ice, and then preserved in −70 °C degrees. Cell extraction and DNA isolation was made with following instructions in *FastDNA™ SPIN Kit for Soil* (MP Biomedicals, Santa Ana, CA). However, small changes were made to protocol for maximizing the amount of bacterial DNA: Tissue Lyser II was used in cell extraction to make sure that all cells broke down, and after Tissue Lyser II, samples rotated at centrifuge twice longer than proposed in the kit protocol. The V1-V3 region of the 16S rRNA gene was amplified using barcoded primers (AGAGTTTGATCMTGGCTCAG;^46^ and GTATTACCGCGGCTGCTG;^47^). PCR-cycles were repeated so that result was good in three separate times, and these samples were pooled. DNA amplification of samples started with 30 s denaturation at 98 °C, followed by 18–35 cycles consisting of denaturation (10 s at 98 °C), annealing (30 s at 65 °C), extension (15 s at 72 °C), and a final extension at 72 °C for 5 min. Paired–end sequencing (2 × 300 bp) with Illumina MiSeq was done at the Institute of molecular medicine Finland (FIMM, University of Helsinki). This procedure was also done for control samples, which were collected from each sampling location and from kits and PCR.

#### Serum samples

Sera were preserved in −70 °C degrees and analyzed later in The Skin and Allergy Hospital (Helsinki University Central Hospital) with Phadiatop Combi^®^ –test (ThermoFisher Scientific). If the result showed higher levels of IgE for airborne or food allergens than the clinical reference, 0.35 kU/L, the allergen-specific analyses were performed for following allergens: birch-, timothy-, and mugwort-pollens; dog-, cat- and horse-dander; house dust mite (*Dermatophagoides pteronyssinus*); outdoor mold (*Cladosporum herbarum*); egg white; milk; cod; wheat; peanut; and soya.

### Bioinformatics

PCR primer sequences were removed with cutadapt v.1.4.2^48^ with maximum error rate of 0.2 and minimum match length of 15. Paired-end reads were joined using PEAR v.0.9.6^49^ with default options and quality trimmed using USEARCH v.8.0^50^ –fastq_filter command with options –fastq_maxee 3, -fastq_minlen 365 and –fastq_maxns 0. OTU clustering and chimera removal was done using USEARCH with default options. The OTU representative sequences were classified and aligned with mothur v. 1.36^51^ using the SILVA rRNA gene database v. 123^52^. Phylogenetic tree was drawn using FastTree v.2.1.8^53^ using the generalized time-reversible model.

All non-bacterial OTUs and contaminant OTUs identified from control samples and were removed prior to any downstream analysis (removed OTUs are in Supplementary Table S1). Samples with library sizes smaller than 3000 reads were removed from the analysis. Thus, 261 subjects were included in downstream analyses. Due to large variation in library sizes, the read counts were normalized using the CSS method from metagenomeSeq package v.1.11^54^ in R v. 3.2.4^55^. All 16S rRNA gene sequences have been deposited in the European Nucleotide Archive (accession no. PRJEB14627; http://www.ebi.ac.uk/ena/data/view/PRJEB14627). The clinical datasets analysed during the current study are available from the corresponding author on reasonable request.

### Land use quantification

Addresses of both the first home after birth and the current home were collected in the questionnaire. Address-information was used for getting coordinates of these locations from which the land use data could be counted. The coverage of the different environmental types around homes were calculated from CORINE2012, a publicly available land cover data (25 m resolution in Finland), using level 2 classifications. Land use data was calculated for different buffers from 250 m to 3 km, but only 3 km buffer is reported here. The land-use data was simplified to its first principal component (as in refs 23 and 38), which tends to capture most variation along a rural-urban land-use gradient.

### Statistical analyses

Phylotypic diversity of the microbiota was calculated as the true diversity of samples (aka., Hill’s diversity;^56^): ^*q*^*D*_*i*_ = (Σ*p*_*i*_^*q*^)^1/(1−*q*)^, where the diversity in sample *i* is the inversed root of the sum of proportional abundances of all species in the sample. The value of *q* determines how much weight is given to differences in species abundances, such that *q* = 0 returns species richness. For microbial samples, it has been shown that *q* needs to be greater than one to obtain reliable estimates of sample diversity^57^. Between-sample similarity (often referred to as beta-diversity, which is misleading;^58^) was quantified using the Bray-Curtis dissimilarity index, which was tested against explanatory variables using multiple regression on distance matrices (MRM, as implemented in the vegan package;^59^).

To investigate the influence of age and living environment on the composition of skin microbiota, we used random forest analysis (RF, as implemented in the randomForest package;^60^), which is an extension of decision tree analysis; from a random subset of the data many decision trees are created, from which the conclusion is then voted. Thus, we asked how well age or environmental land-use could be predicted based on sample microbial composition. As random forest analysis does not allow for several response variables, we used partial least squares regression (PLS, as implemented in the pls package^61^, using the SIMPLS algorithm and leave-one-out cross validation;^62^), to estimate the interaction between age and living environment, predicted by the microbiota. This was done by calculating a PLS prediction for age and land-use separately, and also when both were included as responses in the same model. PLS is a form of principal coordinates regression, where a set of latent variables are generated through iteration to be used as predictors. Variables were scaled to zero mean and unit variance for improved predictions. This did not have a qualitative influence on the results, nor did our choice of the normalisation of raw counts (the effect of different normalization methods is shown in Supplementary Figure S1). We ran the analysis on 1000 OTUs with highest variability to make the analysis more efficient. However, the predictions were practically the same when using the entire data. Based on the inspection of the residual mean squared error of prediction (RMSEP), three components were used in all models. The amount of variance explained (*R*^2^) from each of the three models was then used to partition the fractions of variance predicted by the microbiota (e.g., ref. 63): (A) unique prediction of age, (B) shared prediction between age and land-use, and (C) unique prediction of land-use.

The semi-urban group was included in most downstream analyses even though size of group is small as we wanted to see how group between two extremes (rural and urban) behaves, and all collected data were considered important to report. Immunoglobulin E values were transformed to binary, because of large and clinically uninteresting variation in the lower end. Children were defined sensitized if immunoglobulin E value for summed inhalant or food allergens was more than 1 kU/L. This cut-off value was chosen, because it shows stronger correlation with allergic symptoms and a better differentiation ability than reference cut-off value 0.35 kU/L^38^. The interaction between sensitization and various categorical variables were tested with Pearson’s chi-squared test. Through all statistical tests the p-values less than 0.05 were considered significant.

### Data characteristics

The background and lifestyle of study subjects differs considerably across study areas (Table 3): In cities, most children live in apartments, while in rural areas 86% of children live in single houses (information not provided in Table 3). Smoking is more common among rural parents, and high education (i.e. university level) is more common among urban parents. Rural children have more company in their homes: they are less likely to be the only child in family, and also pet ownership is more common. Rural children have more contact with natural environments: Most rural parents report their children to be in regular contact with dirt, and visiting often (at least once a week) in forests.

The average number of bacterial sequences in skin microbiota samples was 84 915 ranging from 6462 to 452 900. These sequences clustered to a total of 5885 different OTUs. On average, 424 OTUs were found from the skin of subjects, ranging from 101 to 1378. The most common phyla were clearly *Firmicutes, Actinobacteria*, and *Proteobacteria* with proportions of 43.6, 30.0 and 15.6% from all sequences, respectively. At the class level, *Bacilli, Actinobacteria*, and *Gammaproteobacteria* were the most abundant groups, with proportions, 38.7, 29.9, and 9.0, respectively. At genus level, *Streptococcus, Propionibacterium*, and *Micrococcus* counted most of the sequences, with proportions, 25.1, 12.3, and 6.2, respectively. More information about common taxa in different areas and in age groups is provided in Supplementary Figures S2–S7.

### Ethics approval and consent to participate

Study was approved by the Ethics committee of University of Helsinki Central Hospital (permission number: 283/13/03/03/2013). Also, separate permissions were acquired from all municipalities where we sampled children who were enrolled to study either from schools or from child health clinics. The study was performed in accordance with and following the Declaration of Helsinki Principles. Sample collection and all subsequent experimental procedures were conducted in accordance with relevant guidelines and regulations. After study enrolment but prior to sampling, we asked parent or guardian of each child to provide a signed informed consent.

## Additional Information

**How to cite this article:** Lehtimäki, J. *et al*. Patterns in the skin microbiota differ in children and teenagers between rural and urban environments. *Sci. Rep.* **7**, 45651; doi: 10.1038/srep45651 (2017).

**Publisher's note:** Springer Nature remains neutral with regard to jurisdictional claims in published maps and institutional affiliations.

## Supplementary Material

Supplementary Material

## Figures and Tables

**Table 1:** The effect of various factors on the composition of the skin microbiota tested with permutational multivariate analysis of variance.

	DF	MS	*F*	*R*^2^	*P*
Library size	1	0.78	4.89	0.021	0.001
Number of siblings	1	0.38	2.38	0.010	0.001
Pet ownership	1	0.42	2.64	0.011	0.001
Antibiotic use (last 12 mon)	1	0.38	1.16	0.005	0.15
Gender	1	0.46	1.83	0.008	0.001
Sensitization to food allergens	1	0.15	0.95	0.004	0.59
Sensitization to dust allergens	1	0.26	1.61	0.007	0.006
Age	5	0.46	2.87	0.062	0.001
Land-use	2	0.38	2.42	0.021	0.001
Age x Land-use	8	0.17	1.08	0.038	0.061

The effect of most factors is significant, but very small. Age, original library size of a sample and land use around current home seems to be the best predictors of the distances between individual skin microbiotas.

**Table 2:** The differences in the prevalence of sensitization and allergic symptoms between urban and rural environments.

Age	Environment	Sensitized %	Symptomatic %						
		Inhalant	Food	Wheeze	Asthma	Rhinitis	Hayfever	Eczema	Atopic dermatitis
Young	*Urban (n* = *54*)	**9**.**52**	**9**.**52**	24.24	4.54	**35**.**82**	7.46	35.82	4.47
	*Rural (n* = *65*)	**22**.**82**	**30**.**43**	23.23	4.04	**20**.**20**	9.09	44.44	13.13
		**P** = **0**.**053**	**P** = **0**.**004**	P = 1	P = 1	**P** = **0**.**039**	P = 0.93	P = 0.34	P = 0.11
Old	*Urban (n* = *17*)	47.06	23.52	29.41	17.64	**76**.**47**	35.29	47.05	5.88
	*Rural (n* = *52*)	35.29	25.49	26.41	5.66	**39**.**62**	18.86	62.26	18.86
		P = 0.56	P = 1	P = 1	P = 0.29	**P** = **0**.**017**	P = 0.28	P = 0.40	P = 0.36

Young contains children from one to four years old, and Old includes children from eight to fourteen years. The statistically significant differences are shown with bold text. The sum variables were created from questions regarding allergic symptoms such that if answer was ‘yes’ in any of questions focusing on certain symptom-group, then children was classified as symptomatic. Questions considered wheeze, asthma, rhinitis, hay fever, eczema, and atopic dermatitis symptoms; thus, sum variables of all of these symptoms-groups were created.

**Table 3:** The background and lifestyle of children living in different study areas differs considerably.

Study area	% living in apartments	% parents smoking	% mother highly educated	% father highly educated	% having no siblings	% having pet	% contacting dirt regularly	% visiting forest often
*Urban*	95.74	9.57	85.26	67.02	38.94	23.15	45.33	9.45
*Semi*-*urban*	59.09	14.28	90.90	54.54	31.81	45.45	52.63	42.10
*Rural*	3.57	25.17	42.55	22.14	15.60	52.48	76.42	64.75
	P = 2.2e-16	P = 0.0094	P = 2.1e-10	P = 8.8e-11	P = 0.00023	P = 3.8e-05	P = 3.5e-05	P = 9.2e-13

**Table 4:** The number of study subjects in the different age groups and in the different study areas.

		<1 yr	1 yr	2 yrs	4 yrs	8 yrs	14 yrs	*Total*
Ave. age months (s.d.)		4.23 (1.88)	14.37 (2.56)	26.36 (2.87)	49.83 (2.37)	101.85 (3.46)	174.04 (3.60)	61.76 (59.54)
Number of subjects	*Urban*	21	18	26	17	9	12	103
	*Semi*-*urban*	3	4	3	0	2	11	23
	*Rural*	18	30	20	25	29	27	149
	**Total**	42	52	49	42	40	50	275
Female %		55	40	45	64	60	60	53
