# Supplementary material for: Patterns in the skin microbiota differ in children and teenagers between rural and urban environments
Source: Sci Rep. 2017 Mar 31;7:45651. doi: 10.1038/srep45651 (PMC5374497; doi:10.1038/srep45651)
Supplement: Supplementary Material [file srep45651-s1.pdf]

SUPPLEMENTARY MATERIAL

Patterns in the skin microbiota differ in children and teenagers between rural and urban environments

Jenni Lehtimäki[a,1], Antti Karkman[a], Tiina Laatikainen[b], Laura Paalanen[b], Leena von Hertzen[c], Tari Haahtela[c], Ilkka Hanski[d] & Lasse Ruokolainen[a]

[a]Department of Biosciences, University of Helsinki, FI-00014 Helsinki, Finland; [b]National Institute for Health and Welfare, FI-00271 Helsinki, Finland; [c]Skin and Allergy Hospital, Helsinki University Central Hospital, FI-00029 Helsinki, Finland; [d]Deceased in May 2016

[1]To whom correspondence should be addressed: Jenni Lehtimäki, PL 65, FI-00014 Helsinki, Finland, +358504480047, jenni.lehtimaki@helsinki.fi

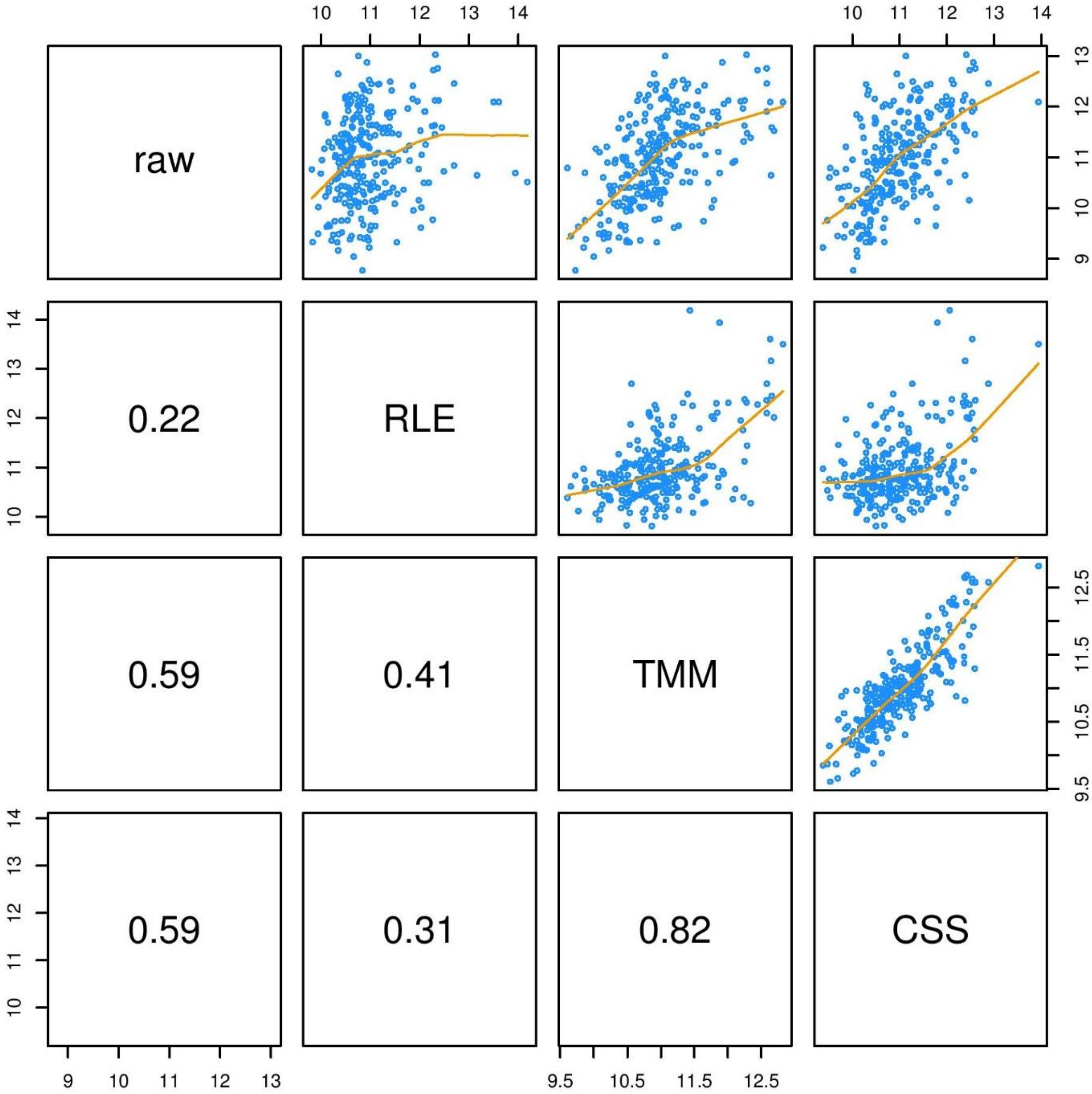

Figure S1. Three different normalizations to raw counts and how they affect library sizes (numbers give Spearman correlation between different data). The lines represent spline smooths to the data. Counts have been log-transformed for clarity. Key: raw = untransformed, RLE = relative log expression normalization [62], TMM = trimmed mean of M-values normalization [63], CSS = cumulative sum normalization [64].

Table S1. Taxa in the table were removed from the microbiota data. Contaminant removal was done separately to dominant and non-dominant arms, because these datasets were handled and sequenced separately.

Dominant arm

Phylum	*Class*	*Order*	*Family*	*Genus*
Proteobacteria	Betaproteobacteria	Burkholderiales	Burkholderiaceae	Ralstonia
Proteobacteria	Betaproteobacteria	Burkholderiales	Burkholderiaceae	Ralstonia
Proteobacteria	Betaproteobacteria	Burkholderiales	Burkholderiaceae	Burkholderia
Proteobacteria	Betaproteobacteria	Burkholderiales	Comamonadaceae	Pelomonas
Proteobacteria	Betaproteobacteria	Burkholderiales	Burkholderiaceae	Burkholderia
Proteobacteria	Betaproteobacteria	Burkholderiales	Burkholderiaceae	Burkholderia
Proteobacteria	Alphaproteobacteria	Rhizobiales	Bradyrhizobiaceae	Bradyrhizobium
Acidobacteria	Acidobacteria	Subgroup_2	unclassified	unclassified
Proteobacteria	Deltaproteobacteria	Oligoflexales	unclassified	unclassified

Non-dominant arm

Phylum	*Class*	*Order*	*Family*	*Genus*
Proteobacteria	Betaproteobacteria	Burkholderiales	Burkholderiaceae	Burkholderia
Proteobacteria	Betaproteobacteria	Burkholderiales	Comamonadaceae	Pelomonas
Proteobacteria	Betaproteobacteria	Burkholderiales	Burkholderiaceae	Burkholderia
Proteobacteria	Betaproteobacteria	Burkholderiales	Burkholderiaceae	Ralstonia
Proteobacteria	Betaproteobacteria	Burkholderiales	Burkholderiaceae	Ralstonia
Proteobacteria	Alphaproteobacteria	Rhizobiales	Phyllobacteriaceae	Mesorhizobium
Proteobacteria	Deltaproteobacteria	Oligoflexales	unclassified	unclassified
Acidobacteria	Acidobacteria	Subgroup_2	unclassified	unclassified
Proteobacteria	Alphaproteobacteria	Rhizobiales	Bradyrhizobiaceae	Bradyrhizobium
Proteobacteria	Gammaproteobacteria	Pseudomonadales	Pseudomonadaceae	Pseudomonas
Proteobacteria	Betaproteobacteria	Burkholderiales	Burkholderiaceae	Burkholderia

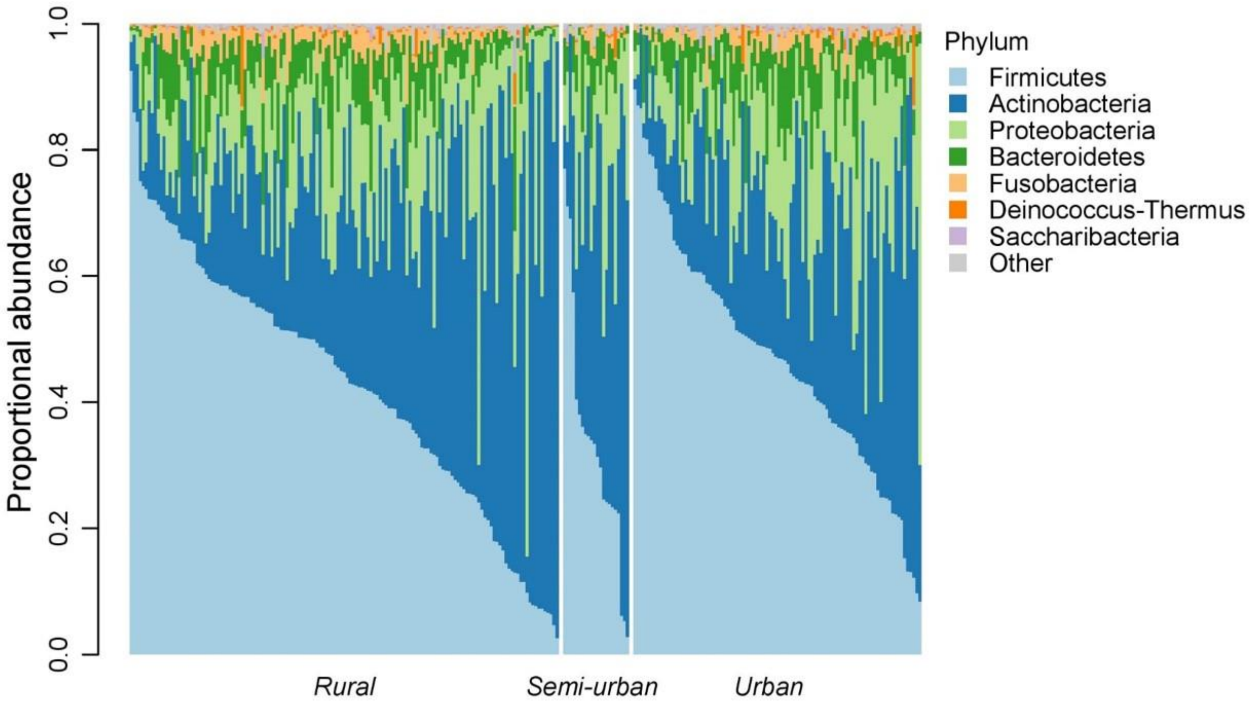

Figure S2. The most abundant taxa on phylum level in the skin microbiota in different study groups.

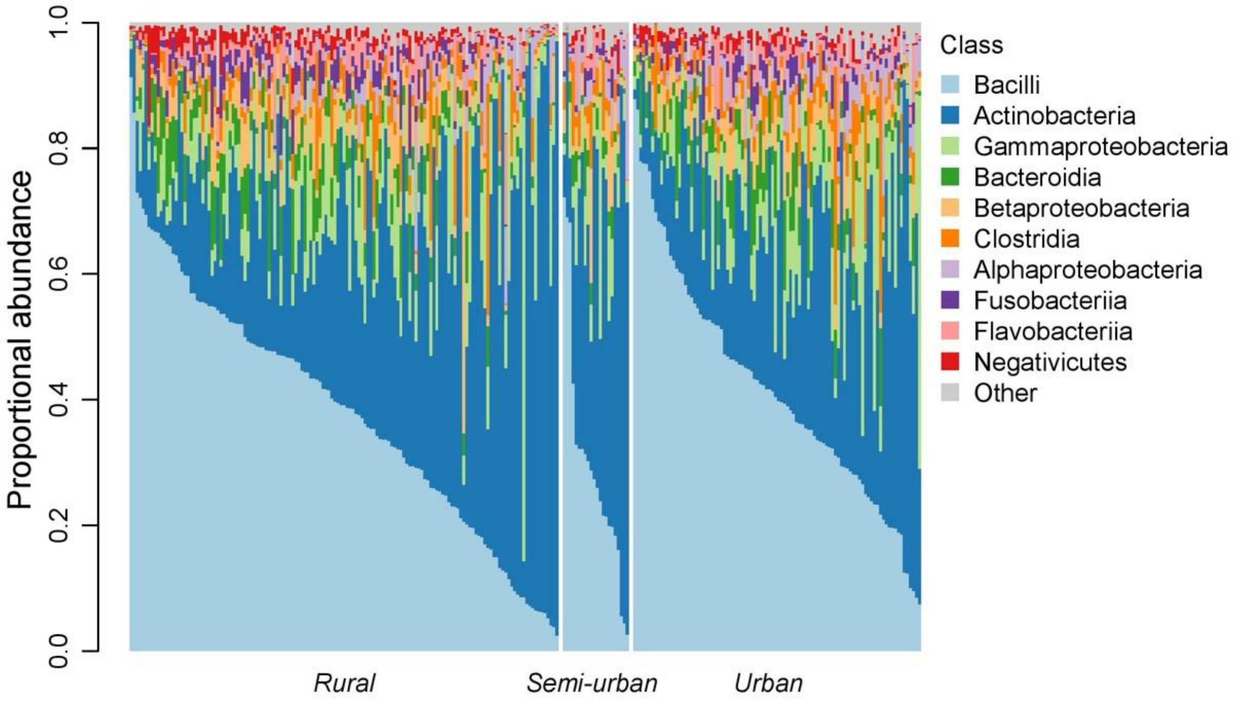

Figure S3. The most abundant taxa on class level in the skin microbiota in different study groups.

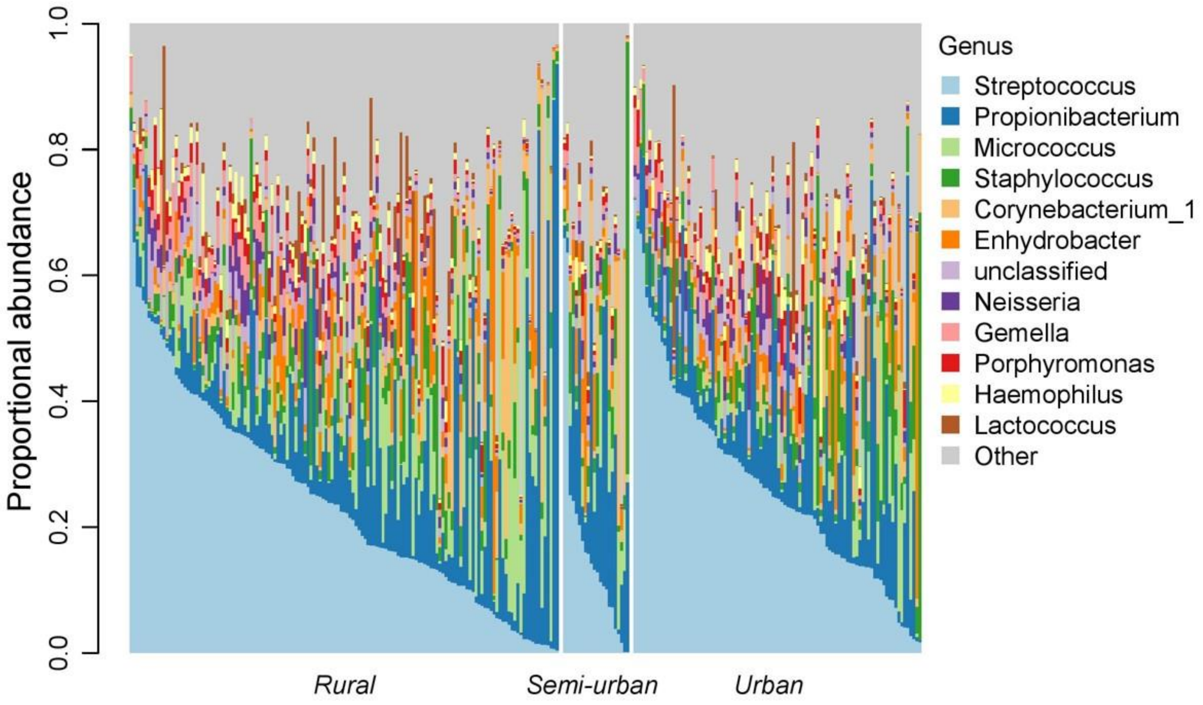

Figure S4. The most abundant taxa on genus level in the skin microbiota in different study groups.

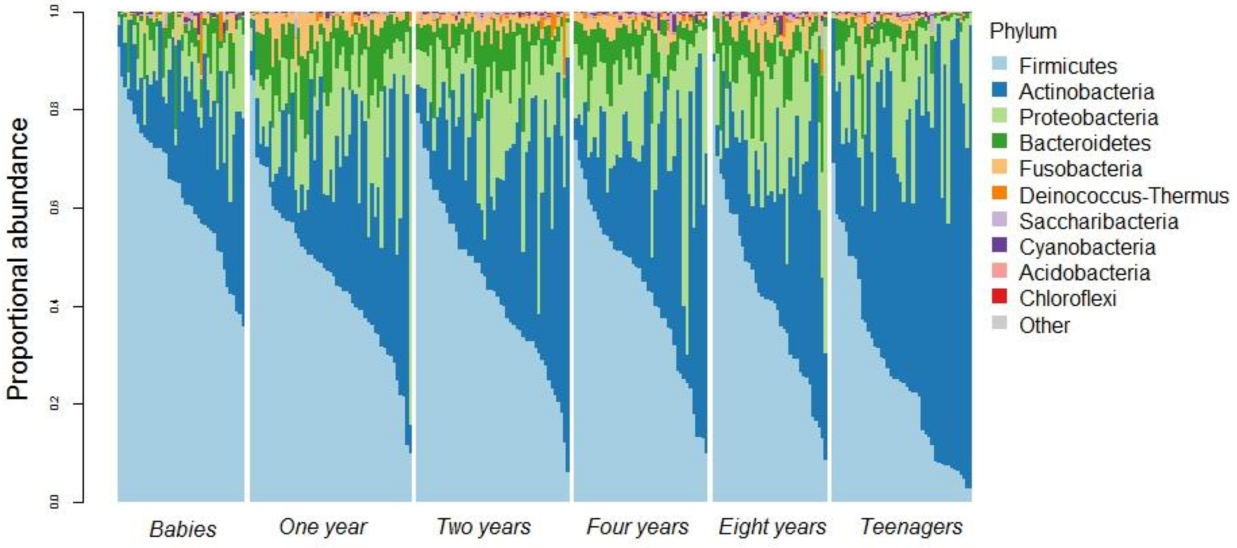

Figure S5. The most abundant taxa on phylum level in the skin microbiota in different age groups.

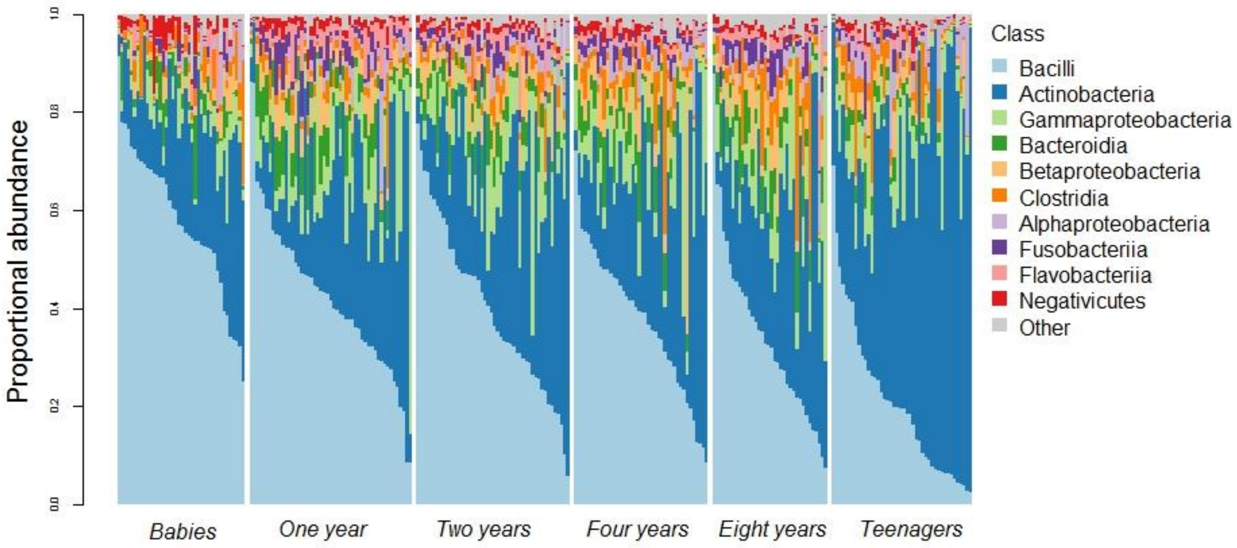

Figure S6. The most abundant taxa on class level in the skin microbiota in different age groups.

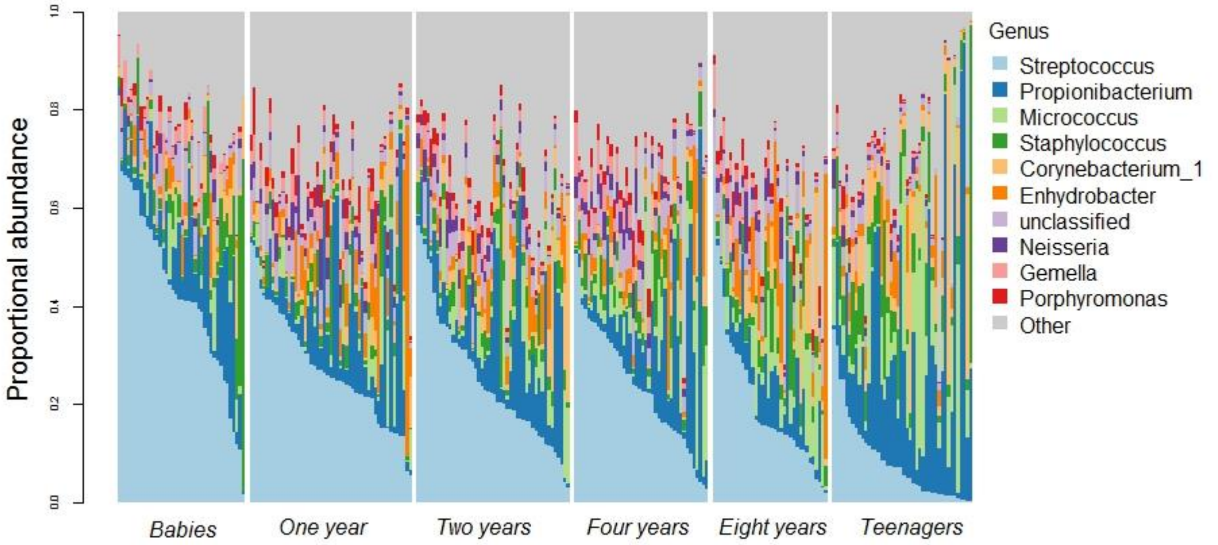

Figure S7. The most abundant taxa on genus level in the skin microbiota in different age groups.

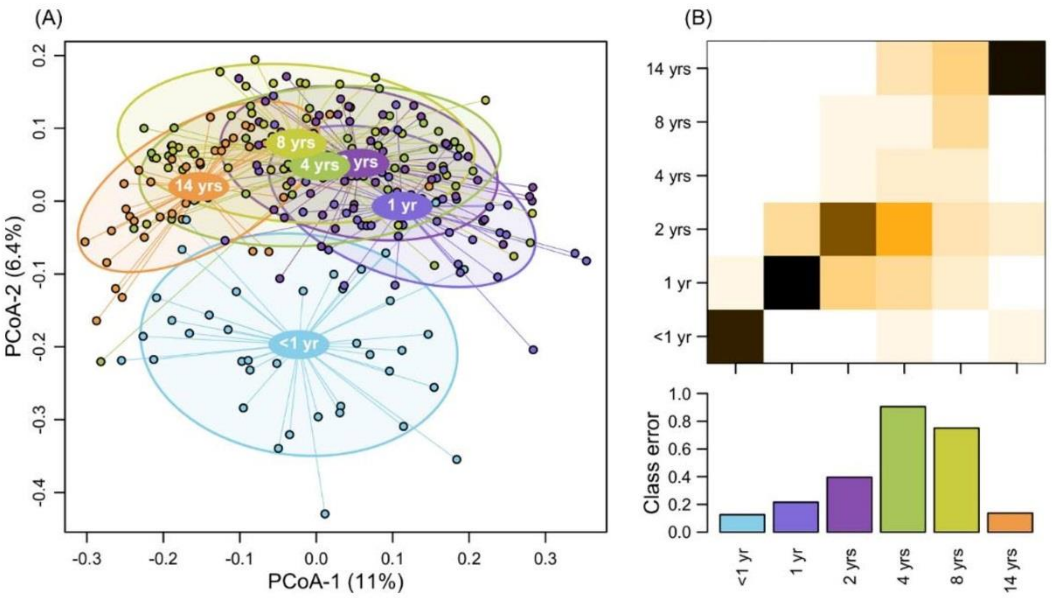

Figure S8. (A) PCoA with Bray-Curtis dissimilarities (based on sqrt-CSS counts). For each group, 75% confidence ellipses are drawn (as implemented in `ordiellipse` in package `vegan` in R). The color key is the same as in panel (B) bottom figure. (B) Random forest (RF) classification of microbiota to age groups. Top: confusion matrix from RF-classification (using 1000 trees): darker colour on the diagonal indicates higher within-group coherence. Bottom: relative class error, indicating the integrity of each class, given the microbiota.

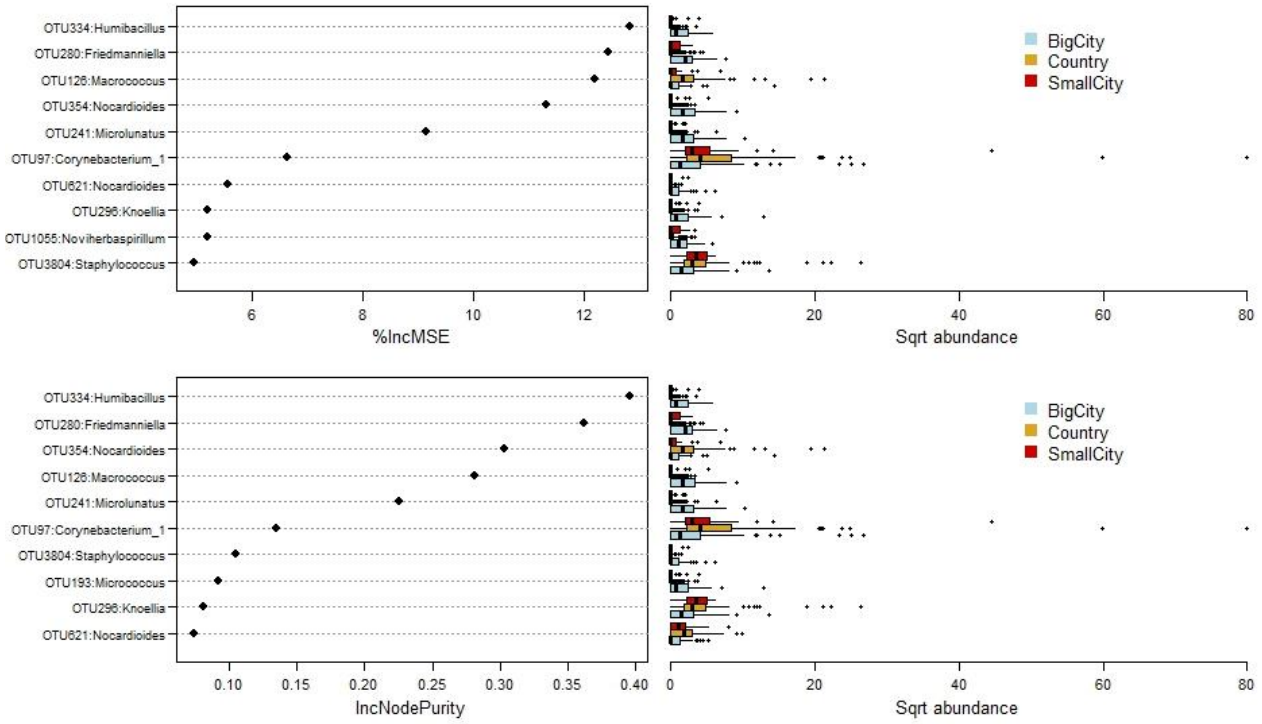

Figure S9. The figure shows the OTUs random forest analysis finds the most significant microbes explaining the patterns.

Table S2. Partitioning of prediction variance using PLS (package pls). The model uses only 1000 OTUs with highest SD (this is faster but the prediction is practically the same as with the entire data). Scaling the predictors to unit variance and zero mean gives a better prediction, but the result is qualitatively the same as with unscaled predictors. The message is that age is quite predictable, given the microbial composition of individuals. While land-use is also fairly well predicted from the microbiota, the predictability depends on age, as suggested by the fact that the prediction of land-use overlaps almost entirely with that of age. RA= Relative abundance, RLE= Relative log expression (package edgeR), CSS= Cumulative sum scaling (package metagenomeSeq).

Normalization	Scaling	Uniq Env	Shared	Uniq Age	Total
none	*unscaled*	0.05	0.32	0.22	0.58
	scaled	0.04	0.43	0.27	0.74
RA	*unscaled*	0.02	0.43	0.24	0.69
	scaled	0.01	0.70	0.15	0.87
RLE	*unscaled*	0.02	0.30	0.29	0.61
	scaled	0.02	0.54	0.27	0.83
CSS	*unscaled*	0.03	0.35	0.20	0.58
	scaled	0.05	0.57	0.26	0.88

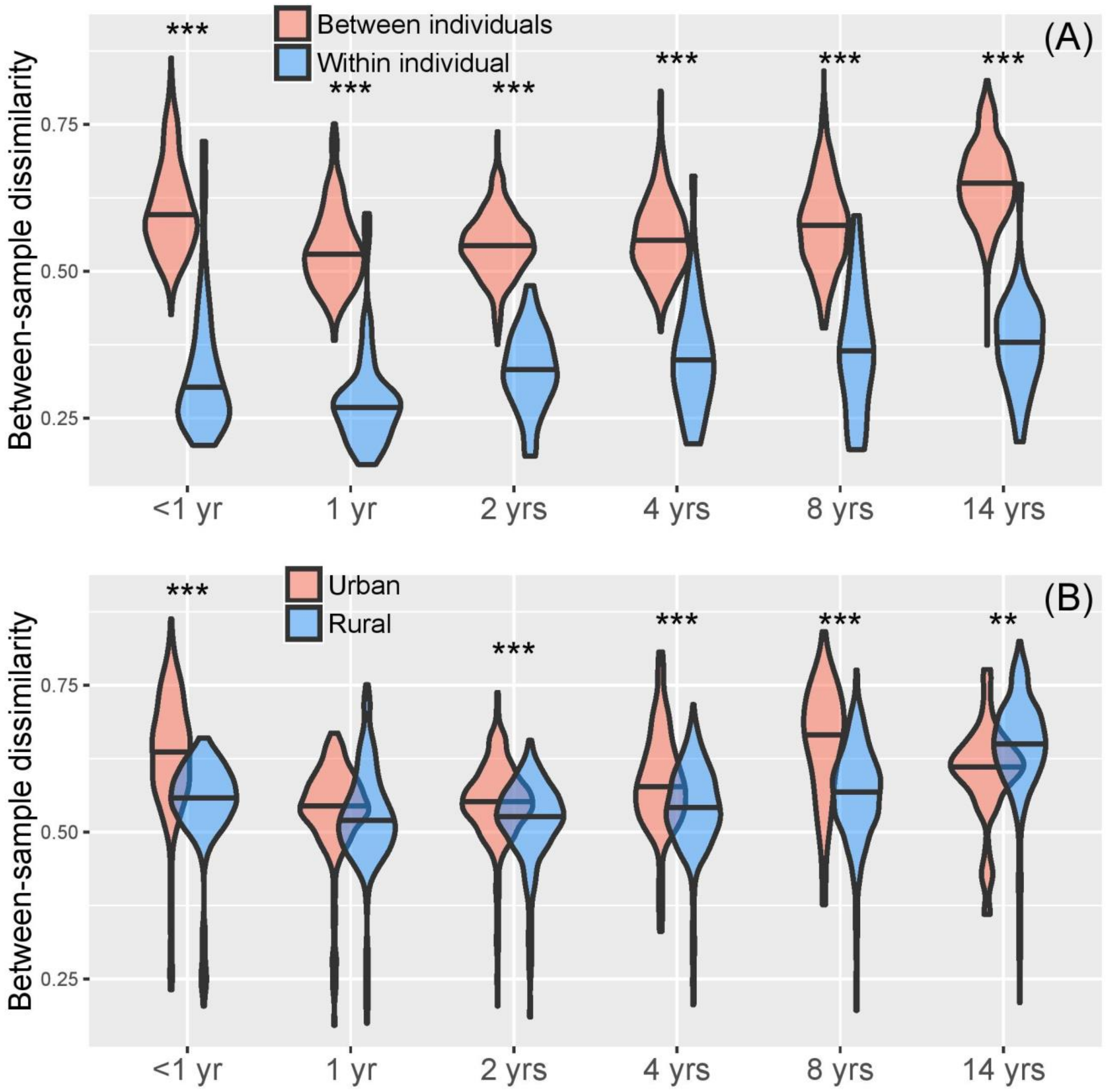

Figure S10. Figures show the Bray-Curtis dissimilarity between the dominant and non-dominant arms in different age groups. Figure (A) shows that in each age group, the intra-individual (blue) dissimilarities are smaller than inter-individual dissimilarities (red). Moreover the intra-individual dissimilarity tends to increase with increasing age. However, less than a year old children are exception from this pattern. Figure (B) shows than in each age group except in teenagers, the intra-individual dissimilarities are smaller in rural children when compared to urban children even though this difference is not significant in one year-old children.

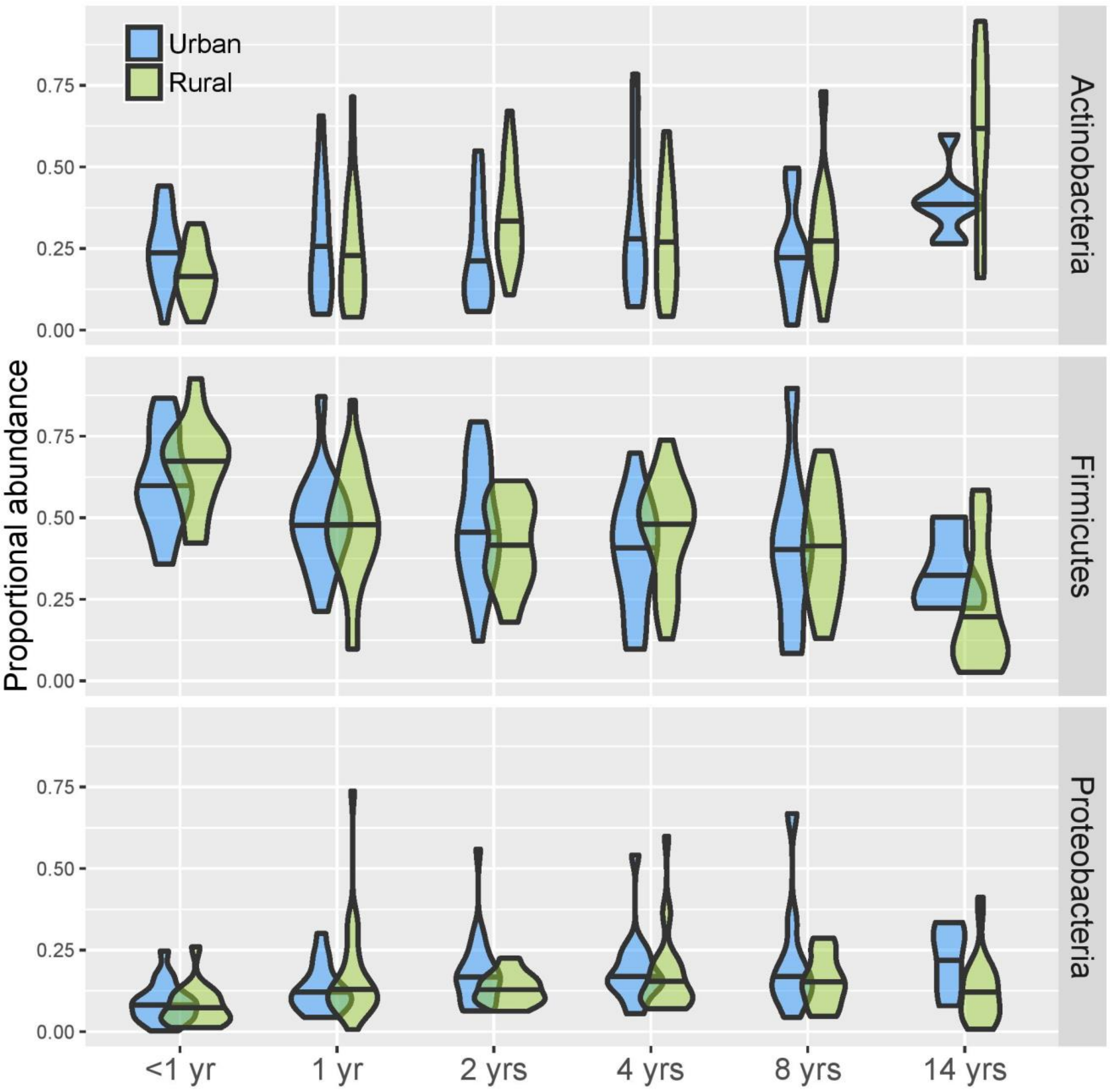

Figure S11. The proportional abundance of Actinobacteria (upper panel), Firmicutes (middle panel), and Proteobacteria (lower panel).

HELSINGIN YLIOPISTO
HELSINGFORS UNIVERSITET
UNIVERSITY OF HELSINKI

Form S1. The questionnaire form for the guardians of less than a year old children.

Patterns in the skin microbiota differ in children and teenagers between rural and urban environments

Less than a year old children

Questionnaire for guardian

1. BASICS

1. Date
2. Birthday
3. Gender
4. Name of the nursery

2. (ALLERGIC) SYMPTOMS

2a. ECZEMA SYMPTOMS

5. Has a child **ever** had an itchy rash?

1 No
2 Yes

6. Has a child **ever** had itchy rash in some of the following sites: *in front of elbows, in back of knees, front side of ankles, buttocks, neck, or around of eyes or ears*?

1 No
2 Yes, constantly.
3 Yes, but it disappears every now and then.

7. Has child **ever** had atopic dermatitis?

1 No
2 Yes, according guardian’s own judgement
3 Yes, diagnosed by a medical doctor

2b. REGURGITATION

8. Does a child regurgitate more than you think is normal?

1 No
2 Yes

9. How many times a child regurgitates daily?

1 1-5 times
2 6-10 times
3 more than 10 times

10. Does a child regurgitate often more than an hour after eating?

1 No
2 Yes

2c. DIARRHEA

11. Have a child had unsual diarrhea which lasts for days?

1 No
2 Yes

2d. SYMPTOMS OF PARENTS

12. Does the biological mother of a child have..

	No	Yes, according own judgement	Yes, diagnosed
..hay fever	☐	☐	☐
..asthma	☐	☐	☐
..atopic dermatitis	☐	☐	☐

13. Does the biological father of a child have..

	No	Yes, according own judgement	Yes, diagnosed
..hay fever	☐	☐	☐
..asthma	☐	☐	☐
..atopic dermatitis	☐	☐	☐

3. FAMILY AND LIFESTYLE

14. How many older siblings a child has?
___ older siblings

15. Which kind of education mother and father of a child has?

	mother	father
Elementary or primary school	☐	☐
Middle or vocational school	☐	☐
High school or college	☐	☐
University	☐	☐

16. Does the guardians of a child or some other people who with child is daily, smoke?

1 No
2 Yes

17. Is a child daily exposed to indoor smoking?

1 No
2 Yes

18. The birth weight of a child was ________________ grams.

19. Was a child breastfed?

1 No
2 Yes, how long? ____

20. How old was a child when supplementary feeding started?
____ months old

21. Do you use unpasteurised, unhomogenised or raw milk in your family?

1 No
2 Yes, regularly.
3 Yes, sometimes.

22. Do you use butter in your family?

1 No
2 Yes, regularly.
3 Yes, sometimes.

23. Has a child received antibiotics?

1 No
2 Yes, how many times? _________

4. LIVING ENVIRONMENT

24. Address of the home of birth:
____

25. Postal code of the home of birth: ________________

26. Current home is in

1 the center of the city
2 the center of the municipality
3 suburban in city
4 conurbation in countryside
5 sparsely populated area

27. Current home is in

1 Apartment house
2 Row house
3 Town house
4 Farm

28. Current home has

1 Own yard
2 Shared yard
3 No yard

29. Does family practice farming currently? (gardening is not counted as a farming)

1 No
2 Yes, full-time
3 Yes, part-time

30. Does family has a dog?

1 No
2 Yes, since year _______________

31. Does family has a cat?

1 No
2 Yes, since year _______________

32. Does family has some other furry pet?

1 No
2 Yes, since year _______________

33. Is a child interacting with pets of other families at least once a week (for example in daycare or in relatives)?

1 No
2 Yes, to which animal? _______________

5. RESPONDENT

34. This form was filled by

1 mother of the child
2 father of the child
3 parents together
4 somebody else, who? ________________________

35. Feedback or more information:

HELSINGIN YLIOPISTO
HELSINGFORS UNIVERSITET
UNIVERSITY OF HELSINKI

Form S2. The questionnaire form for the quardians of 1-4 years old children.

Patterns in the skin microbiota differ in children and teenagers between rural and urban environments

1-4 years old children

Questionnaire for guardian

1. BASICS

1. Date

2. Birthday

3. Gender

4. Name of the nursery

5. Where a child has been in daycare?

	less than a year old	1–3 years	4 years
Home	☐	☐	☐
Outside of home	☐	☐	☐

6. If a child has been in daycare outside of home, in which kind of group a child has been?

	less than a year old	1–3 years	4 years
in group with less than 5 children	☐	☐	☐
in group with 5 to 15 children	☐	☐	☐
in group with more than 15 children	☐	☐	☐

2. ALLERGIC SYMPTOMS

2a. RESPIRATORY SYMPTOMS

7. Has there **ever** been wheezing sound in child's breath?

1 No
2 Yes

8. Has there been wheezing sound in child's breath **during the last twelve months**?

1 No
2 Yes

9. Has a child **ever** had asthma?

1 No
2 Yes, according guardian's own judgement
3 Yes, diagnosed by a medical doctor

10. Has a child taken medicine for treatment of asthma **during the last twelve months**?

1 No
2 Yes
3 A child is not having an asthma

2b. RHINITIS SYMPTOMS

All questions in this part concern the situation when a child **IS NOT having flu or respiratory tract infection!**

11. Has a child **ever** had sneezing, nasal congestion or sniffles when NOT having a flu or respiratory tract infection?

1 No
2 Yes

12. Has a child had sneezing, nasal congestion or sniffles when NOT having a flu or respiratory tract infection **during the last twelve months**?

1 No
2 Yes

13. Has a child had itchy or watery eyes in connection with these rhinitis symptoms **during the last twelve months**?

1 No
2 Yes

14. In which month(s) these rhinitis symptoms occured **during the last twelve months** (you can choose many)?

1 January
2 February
3 March
4 April
5 May
6 June
7 July
8 August
9 September
10 October
11 November
12 December

15. In which age(s) a child has had hay fever symptoms (you can choose many)?

1 Less than a year old
2 One year old
3 Two year old
4 Three year old
5 Four year old
6 A child has never had hay fever

16. If a child has had hay fever symptoms, is hay fever diagnosed by medical doctor?

1 No
2 Yes
3 A child is not having hay fever

2c. ECZEMA SYMPTOMS

17. Has a child **ever** had an itchy rash?

1 No
2 Yes

18. Has a child had an itchy rash **during the last twelve months**?

1 No
2 Yes

19. Has a child **ever** had itchy rash in some of the following sites: *in front of elbows, in back of knees, front side of ankles, buttocks, neck, or around of eyes or ears*?

1 No
2 Yes, constantly.
3 Yes, but it disappears every now and then.

20. Has child **ever** had atopic dermatitis?

1 No
2 Yes, according guardian's own judgement
3 Yes, diagnosed by a medical doctor

2d. SYMPTOMS OF PARENTS

21. Does the biological mother of a child have..

	No	Yes, according own judgement	Yes, diagnosed
..hay fever	☐	☐	☐
..asthma	☐	☐	☐
..atopic dermatitis	☐	☐	☐

22. Does the biological father of a child have..

	No	Yes, according own judgement	Yes, diagnosed
..hay fever	☐	☐	☐
..asthma	☐	☐	☐
..atopic dermatitis	☐	☐	☐

3. FAMILY AND LIFESTYLE

23. How many older and younger siblings a child has?
___ older siblings and
___ younger siblings.

24. Which kind of education mother and father of a child has?

	mother	father
Elementary or primary school	☐	☐
Middle or vocational school	☐	☐
High school or college	☐	☐
University	☐	☐

25. Does the guardians of a child or some other people who with child is daily, smoke?

1 No
2 Yes

26. Is a child daily exposed to indoor smoking?

1 No
2 Yes

27. The birth weight of a child was ________________ grams.

28. Was a child breastfed?

1 No
2 Yes, how long? ____

29. How old was a child when supplementary feeding started?
____ months old

30. Do you use unpasteurised, unhomogenised or raw milk in your family?

1 No
2 Yes, regularly.
3 Yes, sometimes.

31. Do you use butter in your family?

1 No
2 Yes, regularly.
3 Yes, sometimes.

32. Has a child received antibiotics **during the last twelve months**?

1 No
2 Yes, how many times? __________

33. Does a child have following food allergies?

	No	Yes	I don't know
fish	☐	☐	☐
egg	☐	☐	☐
milk	☐	☐	☐
grains	☐	☐	☐
citrus fruits	☐	☐	☐
nuts	☐	☐	☐
apple	☐	☐	☐
carrot	☐	☐	☐

4. LIVING ENVIRONMENT

34. Address of the home of birth:

____

35. Postal code of the home of birth: _________________

36. A child lived in home of birth until the year______ (mark current year if a child is still living in the same apartment).

37. Address of the current home if different than the address of home of birth:

____

38. Postal code of the current home: _________________

39. A child has lived in current home from year __________.

40. Since birth a child has moved..
1 once
2 twice
3 three times
4 more often than three times
5 A child has never moved

41. Current home is in

1 the center of the city
2 the center of the municipality

3 suburban in city
4 conurbation in countryside
5 sparsely populated area

42. Current home is in

1 Apartment house
2 Row house
3 Town house
4 Farm

43. Current home has

1 Own yard
2 Shared yard
3 No yard

44. A child spends time outdoors outside of yard

1 daily
2 almost daily
3 one to three times a week
4 less than once a week

45. Did family practiced farming when child was a year old or younger? (gardening is not counted as a farming)

1 No
2 Yes, full-time
3 Yes, part-time

46. Does family practice farming currently? (gardening is not counted as a farming)

1 No
2 Yes, full-time
3 Yes, part-time

47. Does family has a dog?

1 No
2 Yes, since year _______________

48. Does family has a cat?

1 No
2 Yes, since year ______________

49. Does family has some other furry pet?

1 No
2 Yes, since year ______________

50. Is a child interacting with pets of other families at least once a week (for example in daycare or in relatives)?

1 No
2 Yes, to which animal? ______________

51. Is a child interacting with farm animals (cow, pig, horse, coat, sheep etc.)?

1 daily
2 at least once a week
3 at least once a month
4 at least once a year
5 less than once a year

5. NATURE RELATEDNESS

52. Is family having a summer cottage?

1 No
2 Yes, how many weeks childs spends there yearly? ____________

53. Does a child spend time in countryside with relatives or friends? (if family is not living at countryside)

1 No
2 Yes, how many weeks childs spends there yearly? ____________

54. Mark how often a child visits at following environments:

	Daily	Once a week	Once a month	Few times a year	Less than once a year
forest	☐	☐	☐	☐	☐
park	☐	☐	☐	☐	☐
field	☐	☐	☐	☐	☐
farm	☐	☐	☐	☐	☐
swamp	☐	☐	☐	☐	☐
meadow	☐	☐	☐	☐	☐
sea, lake or river	☐	☐	☐	☐	☐

55. Mark if child does some of the following things more than twice a year (you can choose many):

1 Mushroom picking
2 Berry picking
3 Fishing
4 Hiking
5 Boat trip
6 Hiking overnight

56. When snow is not covering the ground, a child is in contact with natural soils (does not include concrete, gravel, tarmac or sand)?

1 daily
2 weekly
3 rarely
4 never

6. RESPONDENT

57. This form was filled by

1 mother of the child
2 father of the child
3 parents together
4 somebody else, who?______________________

58. Feedback or more information:

HELSINGIN YLIOPISTO
HELSINGFORS UNIVERSITET
UNIVERSITY OF HELSINKI

Form S3. The questtionaire form for the guardians of the 8 and 14 years old children.

Patterns in the skin microbiota differ in children and teenagers between rural and urban environments

8-14 years old children

Questionnaire for guardian

1. BASICS

1. Date

2. Birthday

3. Gender

4. Name of the current school

5. Where a child was in daycare before school?

	less than a year old	1–3 years	4–7 years
Home	☐	☐	☐
Outside of home	☐	☐	☐

6. If a child was in daycare outside of home, in which kind of group a child was?

	less than a year old	1–3 years	4–7 years
in group with less than 5 children	☐	☐	☐
in group with 5 to 15 children	☐	☐	☐
in group with more than 15 children	☐	☐	☐

2. ALLERGIC SYMPTOMS

2a. RESPIRATORY SYMPTOMS

7. Has there **ever** been wheezing sound in child’s breath?

1 No
2 Yes

8. Has there been wheezing sound in child's breath **during the last twelve months**?

1 No
2 Yes

9. Has a child **ever** had asthma?

1 No
2 Yes, according guardian's own judgement
3 Yes, diagnosed by a medical doctor

10. Has a child taken medicine for treatment of asthma **during the last twelve months**?

1 No
2 Yes
3 A child is not having an asthma

2b. RHINITIS SYMPTOMS

All questions in this part concern the situation when a child **IS NOT having flu or respiratory tract infection!**

11. Has a child **ever** had sneezing, nasal congestion or sniffles when NOT having a flu or respiratory tract infection?

1 No
2 Yes

12. Has a child had sneezing, nasal congestion or sniffles when NOT having a flu or respiratory tract infection **during the last twelve months**?

1 No
2 Yes

13. Has a child had itchy or watery eyes in connection with these rhinitis symptoms **during the last twelve months**?

1 No
2 Yes

14. In which month(s) these rhinitis symptoms occured **during the last twelve months** (you can choose many)?

1 January
2 February
3 March
4 April
5 May
6 June
7 July
8 August
9 September
10 October
11 November
12 December

15. In which age(s) a child has had hay fever symptoms (you can choose many)?

1 Less than a year old
2 One year old
3 Two year old
4 Three-four year old
5 Five-six year old
6 Seven-eight year old
7 Nine-ten year old
8 Eleven-twelve year old
9 Thirteen-fourteen year old
10 A child has never had hay fever

16. If a child has had hay fever symptoms, is hay fever diagnosed by medical doctor?

1 No
2 Yes
3 A child is not having hay fever

2c. ECZEMA SYMPTOMS

17. Has a child **ever** had an itchy rash?

1 No
2 Yes

18. Has a child had an itchy rash **during the last twelve months**?

1 No
2 Yes

19. Has a child **ever** had itchy rash in some of the following sites: *in front of elbows, in back of knees, front side of ankles, buttocks, neck, or around of eyes or ears*?

1 No
2 Yes, constantly.
3 Yes, but it disappears every now and then.

20. Has child **ever** had atopic dermatitis?

1 No
2 Yes, according guardian's own judgement
3 Yes, diagnosed by a medical doctor

2d. SYMPTOMS OF PARENTS

21. Does the biological mother of a child have..

	No	Yes, according own judgement	Yes, diagnosed
..hay fever	☐	☐	☐
..asthma	☐	☐	☐
..atopic dermatitis	☐	☐	☐

22. Does the biological father of a child have..

	No	Yes, according own judgement	Yes, diagnosed
..hay fever	☐	☐	☐
..asthma	☐	☐	☐
..atopic dermatitis	☐	☐	☐

3. FAMILY AND LIFESTYLE

23. How many older and younger siblings a child has?
___ older siblings and
___ younger siblings.

24. Which kind of education mother and father of a child has?

	mother	father
Elementary or primary school	☐	☐
Middle or vocational school	☐	☐
High school or college	☐	☐
University	☐	☐

25. Does the guardians of a child or some other people who with child is daily, smoke?

1 No
2 Yes

26. Is a child daily exposed to indoor smoking?

1 No
2 Yes

27. The birth weight of a child was ________________ grams.

28. Was a child breastfed?

1 No
2 Yes, how long? ____

29. How old was a child when supplementary feeding started?
____ months old

30. Do you use unpasteurised, unhomogenised or raw milk in your family?

1 No
2 Yes, regularly.
3 Yes, sometimes.

31. Do you use butter in your family?

1 No
2 Yes, regularly.

3 Yes, sometimes.

32. Has a child received antibiotics **during the last twelve months**?

1 No
2 Yes, how many times? _________

33. Does a child have following food allergies?

	No	Yes	I don't know
fish	☐	☐	☐
egg	☐	☐	☐
milk	☐	☐	☐
grains	☐	☐	☐
citrus fruits	☐	☐	☐
nuts	☐	☐	☐
apple	☐	☐	☐
carrot	☐	☐	☐

4. LIVING ENVIRONMENT

34. Address of the home of birth:

____

35. Postal code of the home of birth: ________________

36. A child lived in home of birth until the year______ (mark current year if a child is still living in the same apartment).

37. Address of the current home if different than the address of home of birth:

____

38. Postal code of the current home: ________________

39. A child has lived in current home from year _________.

40. Since birth a child has moved..
1 once
2 twice
3 three times
4 more often than three times

5 A child has never moved

41. Current home is in

1 the center of the city
2 the center of the municipality
3 suburban in city
4 conurbation in countryside
5 sparsely populated area

42. Current home is in

1 Apartment house
2 Row house
3 Town house
4 Farm

43. Current home has

1 Own yard
2 Shared yard
3 No yard

44. A child spends time outdoors outside of yard

1 daily
2 almost daily
3 one to three times a week
4 less than once a week

45. Did family practiced farming when child was a year old or younger? (gardening is not counted as a farming)

1 No
2 Yes, full-time
3 Yes, part-time

46. Does family practice farming currently? (gardening is not counted as a farming)

1 No
2 Yes, full-time
3 Yes, part-time

47. Does family has a dog?

1 No
2 Yes, since year _______________

48. Does family has a cat?

1 No
2 Yes, since year _______________

49. Does family has some other furry pet?

1 No
2 Yes, since year _______________

50. Is a child interacting with pets of other families at least once a week (for example in daycare or in relatives)?

1 No
2 Yes, to which animal? _______________

51. Is a child interacting with farm animals (cow, pig, horse, coat, sheep etc.)?

1 daily
2 at least once a week
3 at least once a month
4 at least once a year
5 less than once a year

5. NATURE RELATEDNESS

52. Is family having a summer cottage?

1 No
2 Yes, how many weeks childs spends there yearly? _____________

53. Does a child spend time in countryside with relatives or friends? (if family is not living at countryside)

1 No
2 Yes, how many weeks childs spends there yearly? _____________

54. Mark how often a child visits at following environments:

	Daily	Once a week	Once a month	Few times a year	Less than once a year
forest	☐	☐	☐	☐	☐
park	☐	☐	☐	☐	☐
field	☐	☐	☐	☐	☐
farm	☐	☐	☐	☐	☐
swamp	☐	☐	☐	☐	☐
meadow	☐	☐	☐	☐	☐
sea, lake or river	☐	☐	☐	☐	☐

55. Mark if child does some of the following things more than twice a year (you can choose many):

1 Mushroom picking
2 Berry picking
3 Fishing
4 Hiking
5 Boat trip
6 Hiking overnight

56. When snow is not covering the ground, a child is in contact with natural soils (does not include concrete, gravel, tarmac or sand)?

1 daily
2 weekly
3 rarely
4 never

6. RESPONDENT

57. This form was filled by

1 mother of the child
2 father of the child
3 parents together
4 somebody else, who?______________________

58. Feedback or more information:
